# Exhausted phenotype of circulating CD8^+^ T cell subsets in hepatitis B virus carriers

**DOI:** 10.1186/s12865-022-00488-2

**Published:** 2022-04-20

**Authors:** Daixi Jiang, Can Chen, Danying Yan, Xiaobao Zhang, Xiaoxiao Liu, Dong Yan, Dawei Cui, Shigui Yang

**Affiliations:** 1 State Key Laboratory for Diagnosis and Treatment of Infectious Diseases, National Clinical Research Center for Infectious Diseases, Collaborative Innovation Center for Diagnosis and Treatment of Infectious Diseases, The First Affiliated Hospital, Zhejiang University School of Medicine, 79 Qingchun Road, Hangzhou, 310003 China; 2 Department of Blood Transfusion, The First Affiliated Hospital, Zhejiang University School of Medicine, 79 Qingchun Road, Hangzhou, 310003 China; 3 School of Public Health, Zhejiang University, Hangzhou, 310003 China

**Keywords:** HBV, CD8^+^ T cell, CXCR5, Exhaustion, Flow cytometry

## Abstract

**Background:**

Chronic hepatitis B virus (HBV) infection is characterized by the presence of dysfunctional exhausted CD8^+^ T cells that hamper viral control. We investigated the phenotypic heterogeneity of exhausted CD8^+^ T cells in HBV carriers.

**Methods:**

We enrolled 31 HBV carriers and 23 healthy controls (HCs) in our study. Peripheral blood mononuclear cells (PBMCs) were isolated, and flow cytometry was used to determine the phenotypic distribution of CD8^+^ T cell subsets. Expression of cytokines such as TNF-α and IFN-γ was detected by quantitative reverse transcription–PCR, a fluorescence flow cytometry-based immunomicrobead assay and flow cytometry.

**Results:**

There were no significant differences in the baseline characteristics between the 31 HBV carriers and the 23 sex- and age-matched HCs. CD8^+^ T cells exhibited higher levels of inhibitory receptors (TIM3 and PD1) in the HBV carriers than in the HCs (*P* < 0.05); in particular, Tfc cells (CXCR5^+^CD25^−^) expressed higher levels of TIM3 and PD1 than non-Tfc cells in the HBV carriers. In addition, among the subsets of Tc cells, the Tc17 (CXCR5^−^CD25^−^CCR6^+^) subset displayed increased expression of TIM3 and LAG3 in the HBV carriers. Our findings further showed that CD8^+^ T cells produced lower levels of IFN-γ, TNF-α, and Granzyme B. Paired analysis of the Tfc subset and the Tc subset indicated that higher levels of cytokines (IFN-γ and TNF-α) were produced by the Tfc subset in the HBV carriers. Among the Tc subsets, the Tc17 subset produced lower levels of cytokines.

**Conclusion:**

The Tfc subset exhibited an enhanced exhausted phenotype but possessed some functional properties during chronic HBV infection, while the Tc subset showed a lower functional level. The identification of these unique subsets may provide a potential immunotherapeutic target in chronic HBV infection.

**Supplementary Information:**

The online version contains supplementary material available at 10.1186/s12865-022-00488-2.

## Introduction

Hepatitis B virus (HBV) remains a major cause of morbidity and mortality worldwide, causing 1 million deaths annually from complications of persistent infection, liver cirrhosis, and hepatocellular carcinoma [1, 2]. CD8^+^ T cell exhaustion is a state of T cell dysfunction and depletion that plays an important role in the development of chronic HBV infection [3]. When persistent infection is established, the immune response fails to control the virus and can trigger tissue damage, leading to liver cirrhosis and cancer [4]. Accumulating studies support the therapeutic potential of targeting exhausted T cells for the restoration of robust adaptive immune responses [5–7]. However, the pool of exhausted CD8^+^ T cells consists of phenotypically and functionally distinct subsets with distinct levels of responsiveness to intervention [5, 8]. Therefore, the specific subsets of exhausted CD8^+^ T cells during chronic HBV infection should be further identified.

C-X-C motif chemokine receptor type 5 (CXCR5) is expressed by follicular helper CD4 T cells, directing migration to CXCL13-rich B cell follicles and facilitating germinal centre development [9, 10]. Recently, CXCR5 expression was found to define novel subsets of CD8^+^ T cells according to their functional properties; these subsets can be grouped into cytotoxic T (Tc) 1, Tc2. Tc17, regulatory T (Treg), and follicular cytotoxic T (Tfc) subsets [10–12]. The expression of exhaustion markers or inhibitory receptors (IRs) on T cells is a gradual process [7]. Prolonged and/or high coexpression of multiple IRs, such as programmed cell death protein 1 (PD1) and T cell immunoglobulin and mucin domain 3 (TIM3), is a key feature of CD8^+^ and CD4^+^ T cell exhaustion in humans [7, 13]. Several reports have indicated that IR expression is upregulated on HBV-specific CD8^+^ T cells [14–17]. Previous studies indicated that CXCR5^+^ Tfc cells exhibited a reduced state of exhaustion, with lower surface expression of IRs than their counterpart CXCR5^−^ non-Tfc cells during chronic infection [12, 18]. However, in agreement with this concept, a recent study found that circulating CXCR5^+^CD8^+^ T cells expressed high levels of PD1, TIM3, and cytotoxic T lymphocyte antigen 4 (CTLA4) during chronic HBV infection [3]. In addition, restoration of HBV-specific CD8^+^ T cell function by inhibitory receptor blockade in inactive carriers was heterogeneous and linked to T cell differentiation [19]. Due to the heterogeneity of this infectious disease and the complex composition of the CD8^+^ T cell population, no direct comparison of IR expression and effector functions between these novel subpopulations of CD8^+^ T cells during chronic HBV infection has been performed.

In this study, we performed flow cytometric analyses of inhibitory receptor expression, functional properties, and CD8^+^ T cell differentiation marker expression in the peripheral blood mononuclear cells (PBMCs) population of HBV carriers, focusing on the phenotypic heterogeneity of the exhausted CD8^+^ T cells. The identification of this subset may contribute to a better understanding of CD8^+^ T cell exhaustion and provide a potential target in chronic HBV infection.

## Materials and methods

### Patients and samples

All clinical samples were collected at the First Affiliated Hospital of Zhejiang University of Medicine (Hangzhou, China) between December 2020 and June 2021. Thirty-one hepatitis B virus carriers and twenty-three healthy controls (HCs) were enrolled in this study. Serum HBsAg-positive individuals who were retested in 6 months and had normal liver function were included according to the guidelines for the prevention and treatment of chronic hepatitis B (2019 version). In addition, participants with autoimmune liver disease, HAV infection, HCV infection, HDV infection, HEV infection, or other severe or active diseases were excluded. This study was conducted in compliance with the Declaration of Helsinki and was approved by the Ethical Committee of the First Affiliated Hospital of Zhejiang University of Medicine.

### Detection of HBV serological markers and functional biochemical assays

Each HBV-infected participant’s HBsAg, anti-HBs, HBeAg, anti-HBe, and anti-HBc levels were determined in the clinical laboratory of the First Affiliated Hospital of Zhejiang University of Medicine. Serum biochemical markers such as alanine aminotransferase (ALT), aspartate aminotransferase (AST), and alkaline phosphatase (ALP) were detected using an automated analyser (Roche Cobas 8000 c702, Switzerland) according to the manufacturer’s instructions.

### Isolation of peripheral blood mononuclear cells (PBMCs)

Whole blood samples from the donors were collected in heparinized blood collection tubes. The blood samples were diluted by adding the same volume of sterile PBS to the tubes and mixing gently. Then, the mixed samples were overlaid onto the density gradient reagent in the tubes. The samples were centrifuged for 20 min at 800 × *g* and room temperature with the brake function enabled, and the PBMCs layer was identified as the white-coloured interface in the tubes.

### Flow cytometry

PBMCs were washed with PBS and resuspended by adding 0.5 mL of PBS to each tube. Cells were stained with fluorochrome-labelled antibodies (anti-CD4, anti-CD8, anti-CD45RA, anti-CXCR5, anti-CD25, anti-PD1, anti-TIM3, anti-lymphocyte activation gene-3 (LAG3), anti-CTLA4, anti-CCR6, and anti-CXCR3 antibodies) for 20 min at room temperature. The subsets of CD8^+^ T cells were defined according to previous studies [4, 10, 20]. Then, the cells were washed twice with 0.5 mL of PBS. Membrane expression of antibodies on T cells was detected by flow cytometry (Beckman Coulter, Inc., Miami, USA).

### Analysis of effector molecule production

PBMCs were then plated in RPMI 1640 medium containing 10% foetal bovine serum in 96-well U-bottom plates and stimulated with 50 ng/mL PMA (Sigma–Aldrich, USA) and 1 μg/mL ionomycin (PeproTech, USA) for 6 h at 37 °C in the presence of brefeldin A solution (Biolegend, USA) according to the manufacturer’s instructions. Then, the cells were stained with fluorochrome-conjugated antibodies specific for CD4, CD8, CD45RA, CXCR5, CCR6, CD107a, and CXCR3 for 30 min at 4 °C. After washing two times, the cells were fixed for 60 min with a fixation and permeabilization kit (Thermo Fisher Scientific, USA) and were then incubated with the corresponding antibodies (fluorochrome-conjugated anti-IFN-γ, anti-TNF-α, anti-Granzyme B, and anti-FOXP3 antibodies) for 40 min at room temperature for intracellular staining. The stained cells were analysed using a flow cytometer (Cytoflex LX, USA). The data were analysed with CyExpert software. Additional file 1: Fig. S1A, B shows the gating strategy for the subsets of CD8^+^ T cells. The antibodies used in this study are listed in Additional file 1: Table S1.

### Quantitative reverse transcription–polymerase chain reaction (RT–PCR)

Total RNA was extracted from PBMCs using the RNeasy Mini Kit (74104, Qiagen, Germany) according to the manufacturer’s instructions. Thereafter, RNA was reverse transcribed into cDNA, and real-time PCR was performed with a One-step RT–PCR Kit (RR096A, Takara, China). RNA was then detected with an ABI QuantStudio 5 system (Applied Biosystems, USA). Glyceraldehyde 3-phosphate dehydrogenase (GAPDH) was used as the internal control. Relative mRNA expression levels were determined using the 2^−ΔΔCt^ method. The gene-specific primers used in this experiment are listed in Additional file 1: Table S2.

### Detection of cytokines in serum

The serum levels of IL-2, IL-4, IL-6, IL-10, TNF-α, and IFN-γ were detected by a flow cytometry-based fluorescence immunomicrobead assay (CellGene, Inc., Hangzhou, China) according to the manufacturer’s instructions. For this assay, 25 µL of fluorescence detection reagent and 25 µL of immunomicrobeads were added to 25 µL of the samples. The mixed solutions were incubated at room temperature for 2.5 h in the dark. Then, the mixed solutions were washed with 1 mL of PBS and analysed.

### Data analysis

The GraphPad Prism 7 and SPSS 26 statistical analysis software programs were used for statistical analysis of the experimental data, and the results are shown as the means ± SEs. Comparisons between subgroups were performed using the paired samples Wilcoxon test, Mann–Whitney U test, and ANOVA. Tukey’s post-hoc test was applied for comparisons between multiple subgroups. Relationships between different parameters were examined using Spearman correlation analysis. All data were analysed using two-sided tests, and differences with a *P* value < 0.05 were considered significant.

## Results

### CD8^+^ T cells were exhausted in hepatitis B virus carriers

**Fig. 1:**
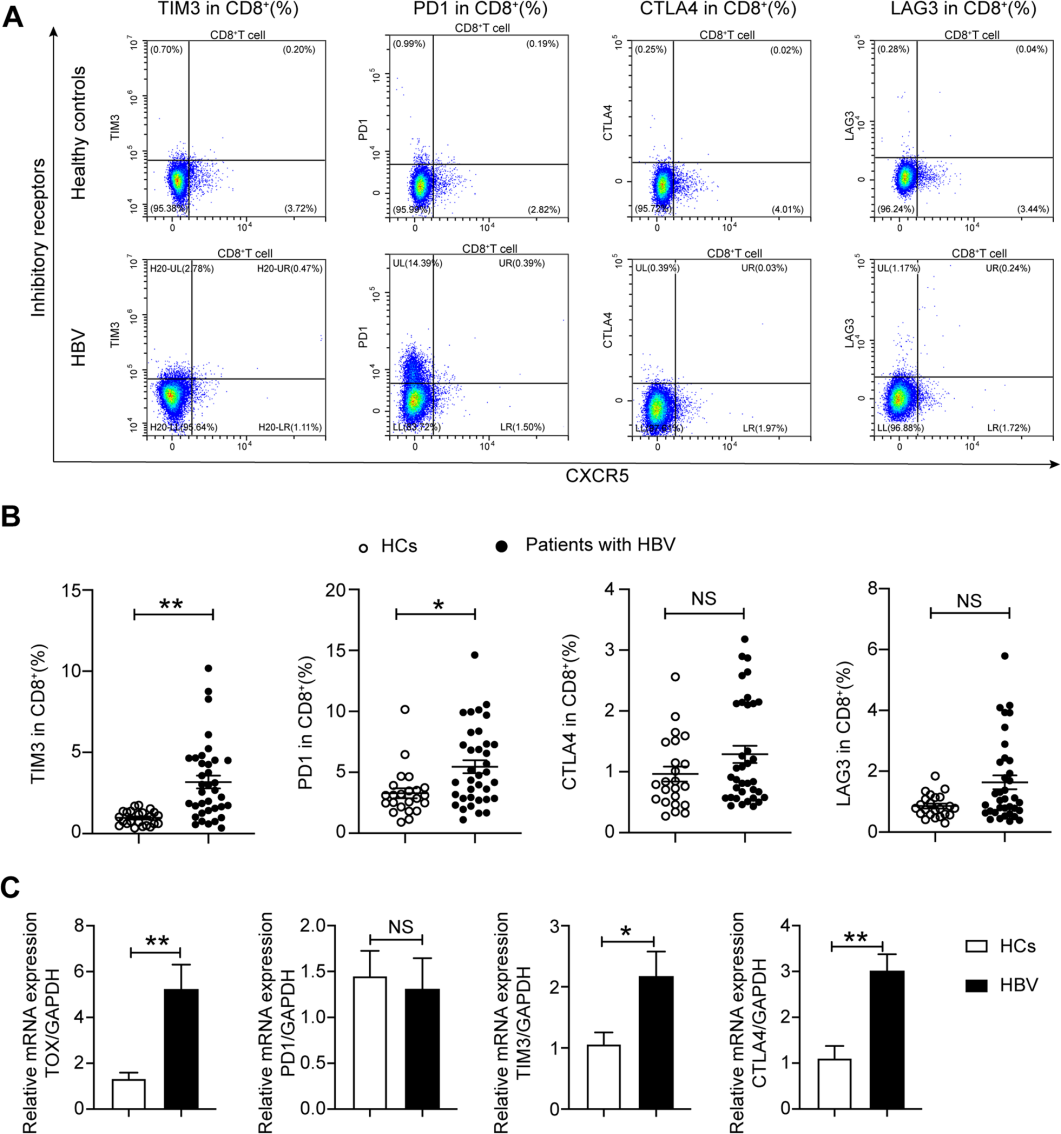
CD8^+^ T cells are exhausted in HBV carriers. **A** Flow cytometric detection of PD1, TIM3, LAG3, and CTLA4 expression on CD8^+^ T cells among PBMCs from HCs and HBV carriers; **B** differential expression of inhibitory receptors in CD8^+^ T cells between HCs and HBV carriers (calculated by the percentage of IR^+^CXCR5^+^CD8^+^ T cells plus the percentage of IR^+^CXCR5^−^CD8^+^ T cells); **C** differential mRNA expression of TOX, PD1, TIM3, and CTLA4 in PBMCs between HCs and HBV carriers. HCs: healthy controls (n = 23), HBV: hepatitis B virus carriers (n = 31); data are presented as the means ± SEs; FDR correction was used to correct the *P* values for comparisons between controls and patients. *P < 0.05 and **P < 0.01

In this study, 31 HBV carriers and 23 HCs were enrolled. The clinical characteristics of the participants are summarized in Table 1. There were no significant differences in the baseline characteristics between the HBV carriers and the HCs (Table 1). Compared with those in HCs, CD8^+^ T cells in HBV carriers showed higher expression levels of PD1, CTLA4, LAG3, and TIM3 to varying degrees (Fig. 1A, B). The frequencies of PD1^+^CD8^+^ and TIM3^+^CD8^+^ T cells were significantly increased in HBV carriers compared with HCs (PD1^+^CD8^+^: 5.45% ± 0.53% vs. 3.29% ± 0.40%, *p* < 0.05; TIM3^+^CD8^+^: 3.17% ± 0.40% vs. 0.96% ± 0.09%, *p* < 0.01, respectively) (Fig. 1B). CD8^+^ T cells expressing multiple IRs were nonsignificantly enriched in HBV carriers (Additional file 1: Fig. S2A). The mRNA expression of the specific transcription factors associated with exhaustion, such as TOX, was higher in the PBMCs of HBV carriers than in those of HCs (Fig. 1C). In addition, the mRNA expression of TIM3 and CTLA4 was increased in the PBMCs of HBV carriers (Fig. 1C).

**Table 1:** Clinical characteristics of patients with chronic HBV infection and healthy controls

Sample size (n)	Chronic HBV infection	Healthy controls
n	31	23
Age (years)	43.65 ± 2.21	43.00 ± 1.69
Gender (male/female)	18/13	13/10
HBsAg (IU/mL)	7469.23 ± 2752.85	–
Anti-HBc (S/CO)	7.73 ± 0.13	–
Anti-HBe (S/CO)	6.58 ± 2.97	–
Anti-HBs (mIU/mL)	1.23 ± 0.62	–
ALT (IU/L)	21.47 ± 1.70	23.00 ± 3.1
AST (IU/L)	21.63 ± 1.18	19.53 ± 0.89
ALP (IU/L)	73.10 ± 3.71	74.09 ± 3.70
Albumin (g/L)	46.26 ± 0.47	45.2 ± 0.58
Globulin (g/L)	26.17 ± 0.51	27.67 ± 1.32
Leukocyte (× 10^9^/L)	5.91 ± 0.29	6.29 ± 0.25
Erythrocyte (× 10^9^/L)	4.91 ± 0.08	4.82 ± 0.10
Lymphocyte (× 10^9^/L)	1.89 ± 0.09	1.99 ± 0.09

*HBsAg* hepatitis B virus surface antigen, *HBeAg* hepatitis B virus e antigen, *ALT* alanine aminotransferase, *AST* aspartate transferase, *ALP* alkaline phosphatase, *NA* not applicable

### CXCR5^+^CD8^+^ T cells exhibited a more suppressive phenotype than their CXCR5^−^ counterparts in HBV carriers

**Fig. 2:**
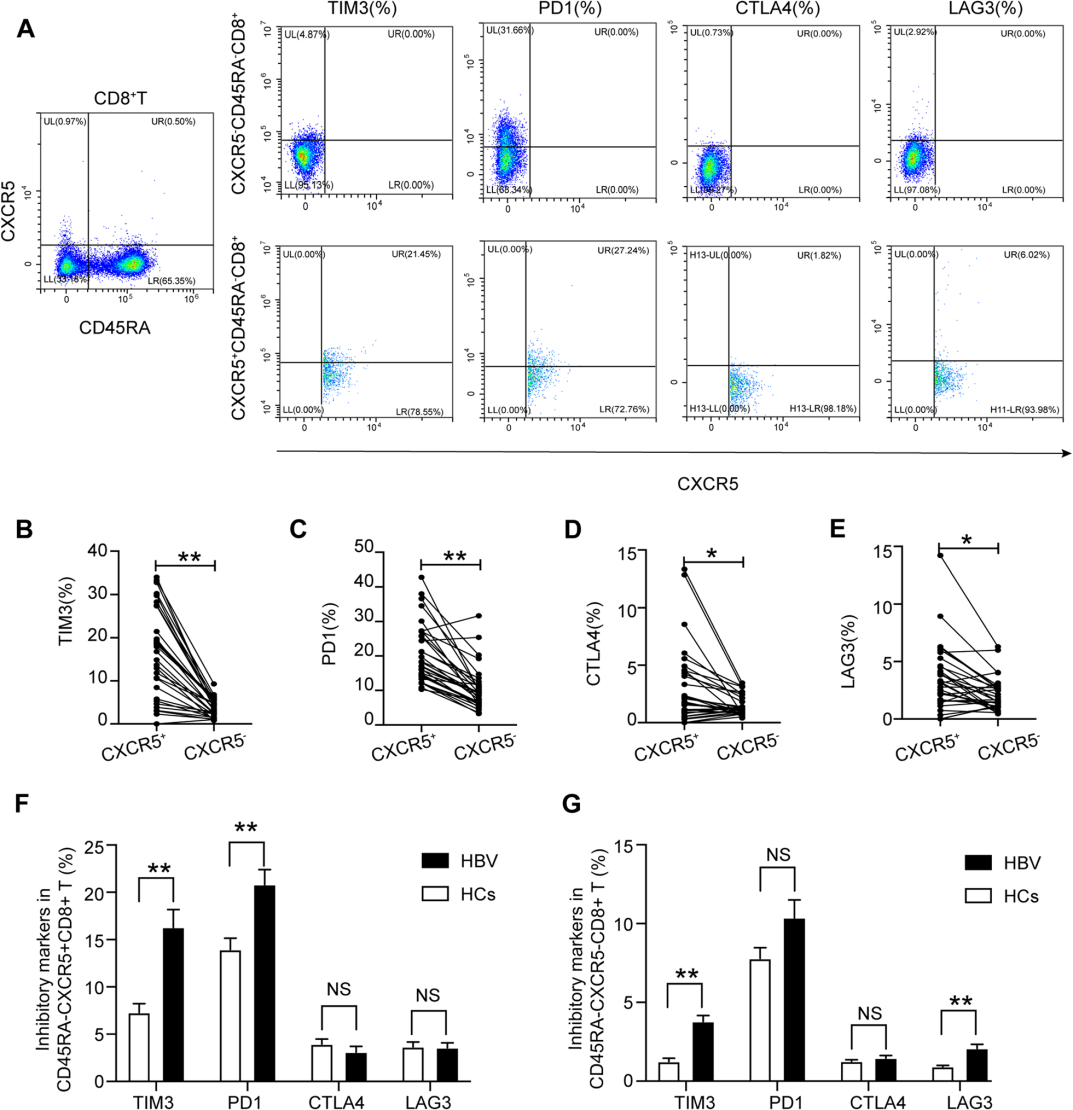
CXCR5^+^CD8^+^ T cells exhibit a more suppressive phenotype than their CXCR5^−^ counterparts in HBV carriers. **A** Flow cytometric detection of PD1, TIM3, LAG3, and CTLA4 on CXCR5^+^CD8^+^ T cells and CXCR5^−^CD8^+^ T cells in HBV carriers; **B**–**E** paired analysis of PD1, TIM3, LAG3, and CTLA4 expression on CXCR5^+^CD8^+^ T cells and CXCR5^−^CD8^+^ T cells in HBV carriers; **F**–**G** differential expression of PD1, TIM3, LAG3, and CTLA4 in CXCR5^+^CD8^+^ T cells and CXCR5^−^CD8^+^ T cells between HCs and HBV carriers. HCs: healthy controls (n = 23), HBV: hepatitis B virus carriers (n = 31); data are presented as the means ± SEs; *P < 0.05 and **P < 0.01

To further identify the subsets expressing suppressive receptors, we tested IR expression on circulating CD45^−^CD8^+^ T cells by flow cytometry. Paired analysis of the CXCR5^+^CD8^+^ T cell group and the CXCR5^−^CD8^+^ T cell group indicated that the expression of the aforementioned IRs was higher on the surface of CXCR5^+^CD8^+^ T cells than CXCR5^−^CD8^+^ T cells in HBV carriers (Fig. 2A). TIM3 expression showed a significant increase in CXCR5^+^CD8^+^ T cells compared to CXCR5^−^CD8^+^ T cells (16.31% ± 1.86% vs. 3.79% ± 1.60%) (Fig. 2B). We found that PD1 expression was significantly increased on the surface of CXCR5^+^CD8^+^ T cells compared to CXCR5^−^CD8^+^ T cells (20.83% ± 1.57% vs. 10.37% ± 1.34%) (Fig. 2C). CTLA4 and LAG3 expression was also elevated slightly in CXCR5^+^CD8^+^ T cells (Fig. 2D, E). The expression of PD1 and TIM3 was higher on the surface of CXCR5^+^CD8^+^ T cells in HBV carriers (Fig. 2F), while the expression of TIM3 and LAG3 was higher on the surface of CXCR5^−^CD8^+^ T cells in HBV carriers (Fig. 2G) than in HCs.

### Enhanced expression of inhibitory receptors on follicular cytotoxic T (Tfc) cells in HBV carriers

**Fig. 3:**
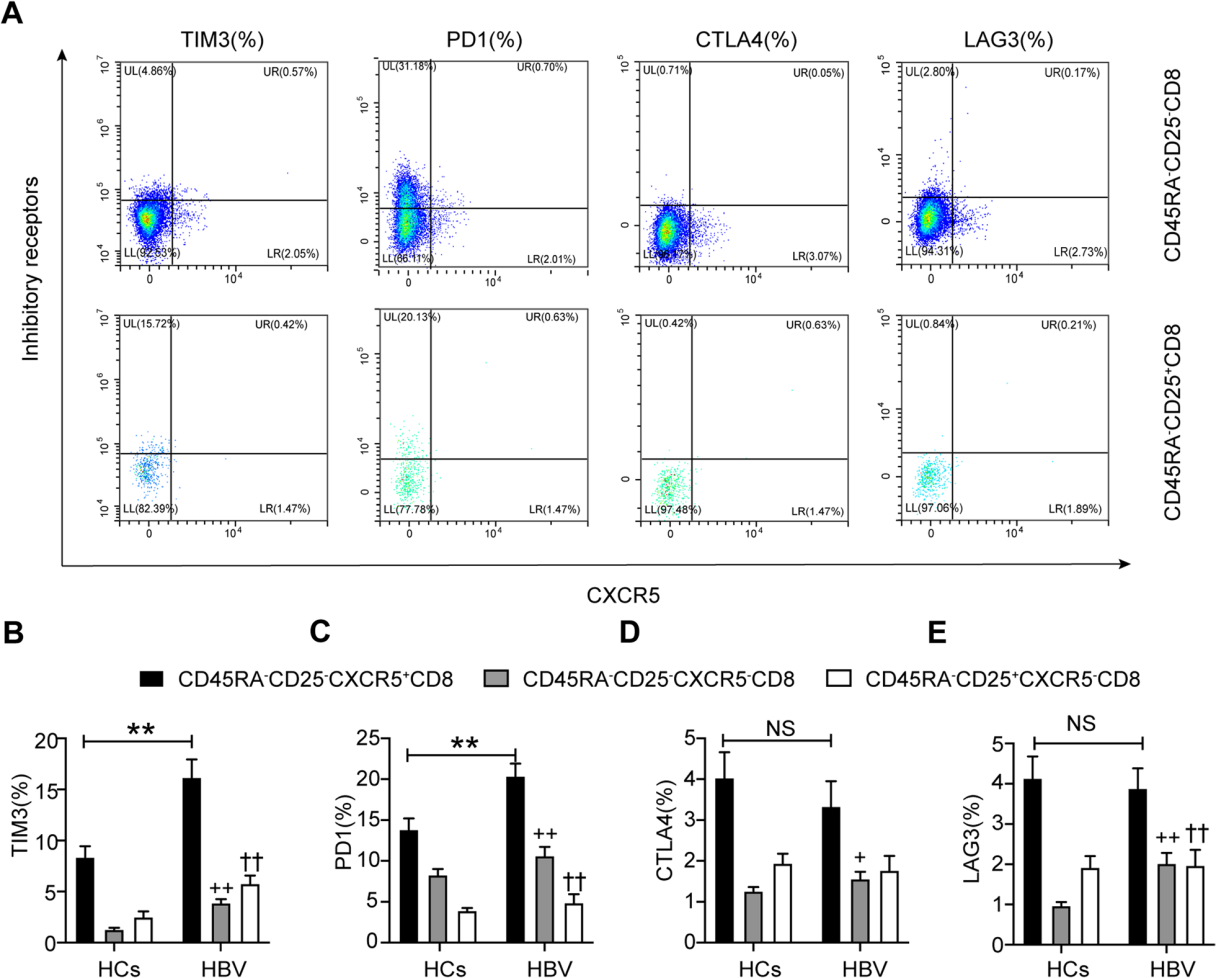
Enhanced expression of inhibitory receptors on Tfc cells in HBV carriers. **A** Flow cytometric detection of PD1, TIM3, LAG3, and CTLA4 on Tfc and Tc/Treg cells in HBV carriers; **B**–**E** differential expression of PD1, TIM3, LAG3, and CTLA4 on Tfc, Tc, and Treg cells in HBV carriers. Tfc: follicular cytotoxic T cell (CXCR5^+^CD25^−^); Treg: regulatory T cell (CXCR5^−^CD25^+^); Tc: cytotoxic T cell (CXCR5^−^CD25^−^); HCs: healthy controls (n = 23), HBV: hepatitis B virus carriers (n = 31); data are presented as the means ± SEs; **P < 0.01; ^††^*P* < 0.01, Tfc versus Treg; ^++^*P* < 0.01, Tfc versus Tc; ^+^*P* < 0.05, Tfc versus Tc

To distinguish the CD8^+^ Treg subsets, we detected circulating CD25^+^CD8^+^ T cells in HCs and HBV carriers (Fig. 3A). We found that surface expression of PD1, TIM3, and LAG3 was significantly increased in the circulating Tfc (CXCR5^+^CD25^−^CD8^+^) subset compared to the CD8^+^ Treg (CXCR5^−^CD25^+^CD8^+^) subset in HBV carriers (Fig. 3B–E). Additionally, the expression levels of PD1, TIM3, CTLA4, and LAG3 were higher in the Tfc subset than in the Tc (CXCR5^−^CD25^−^CD8^+^) subset (Fig. 3B–E). We compared the expression of inhibitory receptors on circulating Tfc cells between HCs and HBV carriers and found that the expression of PD1 and TIM3 was higher on Tfc cells in HBV carriers than in HCs (Fig. 3B, C). However, the difference in CTLA4 and LAG3 expression on Tfc cells between HBV carriers and HCs was nonsignificant (Fig. 3D, E).

### CD8^+^ cytotoxic T cells showed heterogeneity in the suppressive phenotype in HBV carriers

**Fig. 4:**
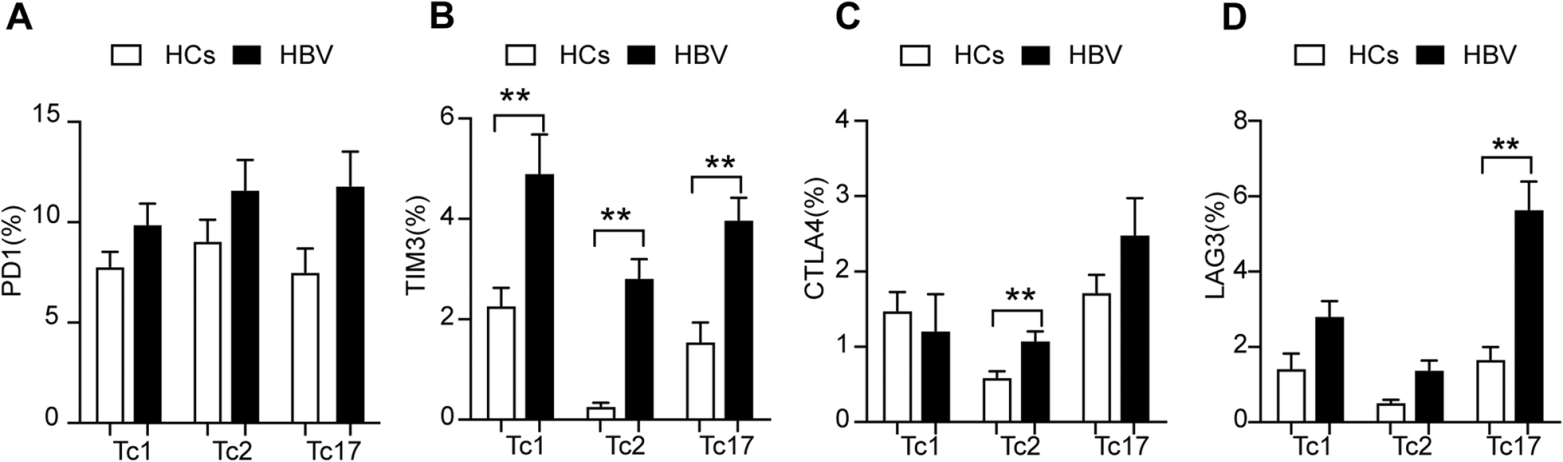
Tc subsets showed heterogeneity in the suppressive phenotype in HBV carriers. **A** PD1 expression on Tc1, Tc2 and Tc17 cells; **B** TIM3 expression on Tc1, Tc2 and Tc17 cells; **C** CTLA4 expression on Tc1, Tc2 and Tc17 cells; **D** LAG3 expression on Tc1, Tc2 and Tc17 cells. HCs: healthy controls (n = 23), HBV: hepatitis B virus carriers (n = 31); data are presented as the means ± SEs; **P < 0.01

The terminal differentiation fates of Tc cells include CD8^+^ Tc1, Tc2, and Tc17 cells. In this study, we found that PD1, TIM3, CTLA4, and LAG3 were upregulated to different degrees on the surface of Tc17 cells in HBV carriers compared with HCs, and the differences in TIM3 and LAG3 expression were significant (Fig. 4A–D). In HBV carriers, the expression levels of LAG3 and CTLA4 were higher on the surface of Tc17 cells than on the surface of Tc2 cells (LAG3: 5.66% ± 0.73% vs. 1.40% ± 0.24%, *p* < 0.01; CTLA4: 2.49% ± 0.48% vs. 1.09% ± 0.12%, *p* < 0.05) (Fig. 4C, D).

### Correlations between circulating subsets of exhausted Tfc/Tc17 cells and the serum/mRNA levels of cytokines in PBMCs of HBV carriers

**Fig. 5:**
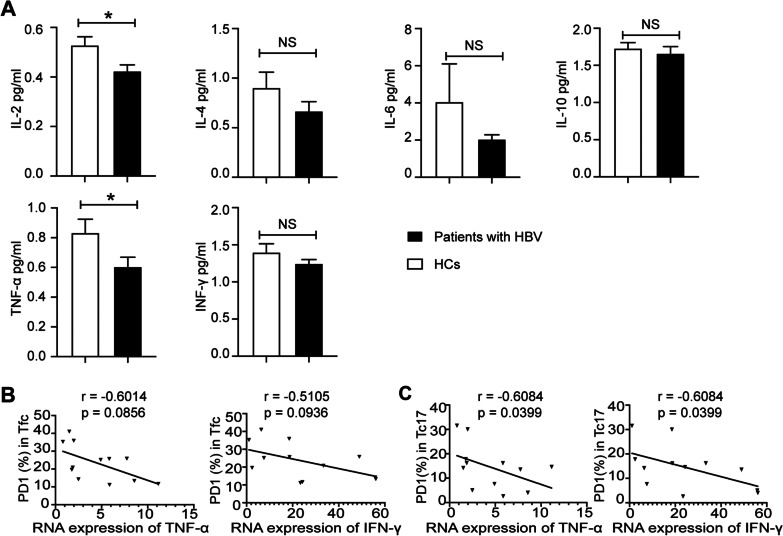
The serum/mRNA levels of cytokines in PBMCs from HBV carriers. **A** Serum levels of cytokines as measured by the flow cytometry-based fluorescence immunomicrobead assay; **B** correlations between the frequencies of PD^+^ Tfc/Tc17 cells and the TNF-α expression level (n = 10); **C** correlations between the frequencies of PD^+^ Tfc/Tc17 cells and the IFN-γ expression level (n = 10). HCs: healthy controls, HBV: hepatitis B virus; data are presented as the means ± SEs; FDR correction was used to correct the *P* values for multiple testing. *P < 0.05

The serum levels of IL-2, IL-4, IL-6, IL-10, TNF-α, and IFN-γ were lower in HBV carriers to varying degrees than in their counterpart HCs. The differences in IL-2 and TNF-α serum levels between the two groups were significant (Fig. 5A). However, the frequencies of PD1^+^ and TIM3^+^ Tfc/Tc17 cells were not related to the serum levels of cytokines in HBV carriers (Additional file 1: Fig. S2B, C). The total RNA expression levels of TNF-α/IFN-γ in PBMCs were also measured. The frequency of PD^+^ Tfc cells was negatively correlated with the RNA expression levels of TNF-α and IFN-γ in HBV carriers without statistical significance (Fig. 5B). However, the frequency of PD1^+^ Tc17 cells was negatively correlated with the RNA expression levels of IFN-γ and TNF-α in HBV carriers (Fig. 5C). The frequencies of PD1^+^, TIM3^+^, LAG3^+^, and CTLA4^+^ Tfc/Tc17 cells were negatively correlated with the serum HBsAg level in HBV carriers, but the difference was nonsignificant (Additional file 1: Table S3).

### The Tfc subset possessed stronger functional properties than the Tc subset in HBV carriers

**Fig. 6:**
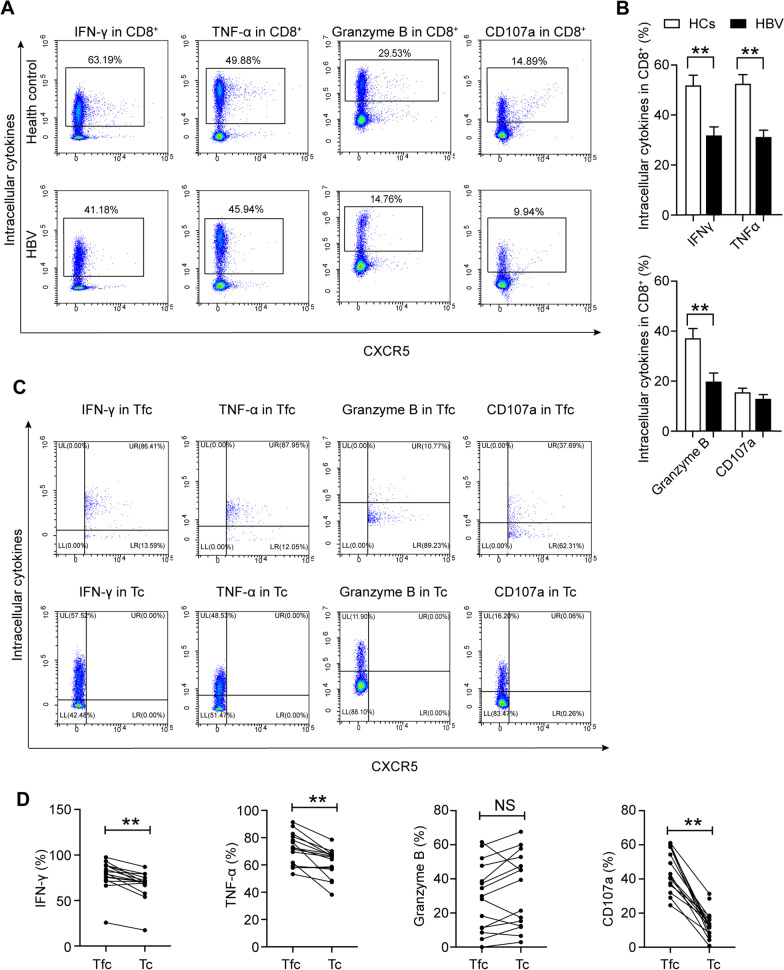
The Tfc subset possessed higher effector functions than the Tc subset in HBV carriers. **A** Flow cytometric detection of IFN-γ, TNF-α, Granzyme B, and CD107a expression on CD8^+^ T cells among PBMCs from HCs and HBV carriers; **B** differential production of effector cytokines by CD8^+^ T cells between HCs (n = 15) and HBV carriers (n = 15); **C** flow cytometric detection of effector cytokines in the Tfc and Tc subsets in HBV carriers; **D** paired analysis of cytokine expression in the Tfc and Tc subsets in HBV carriers. HCs: healthy controls, HBV: hepatitis B virus, Tfc: follicular cytotoxic T cells (CXCR5^+^FOXP3^−^), Tc: cytotoxic T cells (CXCR5^−^FOXP3^−^); data are presented as the means ± SEs; **P < 0.01

We further analysed the functional properties of CD8^+^ T cells on a per-cell basis in HBV carriers and HCs. CD8^+^ T cells from HCs produced higher levels of TNF-α, IFN-γ, and Granzyme B than those from HBV carriers (Fig. 6A, B). Compared with the counterpart subset in the HCs group, the Tc subset in patients with HBV produced lower levels of TNF-α, IFN-γ, and Granzyme B (decreases of 12.18%, 7.13%, and 18.20%, respectively) (Additional file 1: Fig. S2D). The difference in TNF-α production in the Tc subset between the healthy controls and patients was significant. The production of TNF-α, IFN-γ, and Granzyme B in the Tfc subset was also decreased to some degree in patients with HBV (decreases of 7.83%, 4.02%, and 20%, respectively) (Additional file 1: Fig. S2E). However, there were no significant differences in the production of cytokines by Tfc cells between the patients and healthy controls. Compared to the paired Tc subset, the Tfc subset produced significantly higher levels of TNF-α, IFN-γ, and CD107a but a similar level of Granzyme B in HBV carriers (Fig. 6C, D). Among the TC subsets, the Tc17 subset, with higher expression of inhibitory receptors, exhibited lower functional properties (TNF-α, granzyme B, and CD107a production) in HBV carriers (Additional file 1: Fig. S2F).

## Discussion

Chronic HBV infection is characterized by the presence of dysfunctional exhausted CD8^+^ T cells that are unable to control viral infection [21]. In this study, we provide insight into the expression of IRs combined with the functional properties of the circulating CD8^+^ T cells in HBV carriers and healthy controls. We found that the subsets of CD8^+^ T cells, especially Tfc cells, expressed higher levels of IRs, such as PD1 and TIM3, than the subsets of non-Tfc cells in HBV carriers. In addition, in the subsets of non-Tfc cells, the Tc17 subset displayed increased expression of IRs, which are considered hallmarks of T cell exhaustion. CD8^+^ T cells produced lower levels of effector cytokines involved in cytotoxicity; however, the Tfc subset possessed some functional properties during chronic HBV infection.

Our study showed upregulation of inhibitory receptor expression on the surface of circulating CD8^+^ T cells in patients with chronic HBV infection, which was in complete agreement with several reports [14–17]. However, we observed significantly higher expression of PD1 and TIM3 than of CTLA4 or LAG3 on CD8^+^ T cells, suggesting that PD1 and TIM3 might have a greater contribution to the T cell dysfunction observed during HBV infection. TOX is a critical factor for the normal progression of T cell dysfunction and the maintenance of exhausted T cells during chronic infection [22]. Exhausted CD8^+^ T cells exhibit a transcriptional program distinct from that of functional effector or memory CD8^+^ T cells, characterized, for example, by the expression of PD1, TIM3, and CTLA4 [23]. Our mRNA expression data indicated that circulating lymphocytes were exhausted in the patients with HBV. Then, we further identified the subsets of CD8^+^ T cells displaying indications of severe exhaustion, as indicated by the expression of IRs. Novel subsets of CD8^+^ T cells are defined by CXCR5 expression according to their functional properties [10]. CXCR5^+^CD8^+^ T cells have been found in humans; however, no consensus with regard to their function and properties has been reached. CD8^+^ T cells include the memory T cell (CD45A^−^) and the naive/effector T cell (CD45RA^+^) populations [24–26]. In our study, we observed higher expression of IRs in CXCR5^+^CD8^+^ T cells than in their counterparts in the CD45RA^−^ subset. The increase in PD1 and TIM3 expression in CXCR5^+^CD8^+^ T cells was significantly greater than that in CXCR5^−^CD8^+^ T cells in patients with chronic HBV infection relative to healthy controls. The result was similar when we further eliminated the confounding effect of the CD25^+^CXCR5^+^CD8^+^ T cells among the CXCR5^+^CD8^+^ T cells. We observed that the subset of Tfc cells (CD25^−^CXCR5^+^CD8^+^) with memory potential might be one of the major subsets that display an increased state of exhaustion during chronic HBV infection. However, a previous study also demonstrated that CXCR5^+^PD1^+^CD8^+^ T cells are precursors of exhausted CD8^+^ T cells that sustain the population of virus-specific CD8^+^ T cells in mice with chronic LCMV infection [27]. In addition, CXCR5^+^PD1^+^CD8^+^ T cells have been shown to be memory‐like cells, but in contrast to “classical” exhausted PD1^+^ effector T cells, they retain full functional capability [18, 27–29]. In our study, the number of CD45RA^−^Tfc cells with exhaustion markers tended to be inversely correlated with HBV antigen loads; however, the correlation was not significant. It seems that the exhausted Tfc memory cells might have antiviral activity. However, the acquisition of the dysfunctional phenotype appears to be unaffected by the relative antigen load in hepatocytes (even very small amounts of HBV antigens can trigger severe T cell dysfunction) [30]. A previous study also indicated that the exhausted phenotype results from a differentiation process in which T cells stably adjust their effector capacity to the needs of chronic infection [31]. Overall, our results indicated that Tfc cells were the main cell type exhibiting the exhausted phenotype during chronic HBV infection, and investigation of the effector function of Tfc subsets is warranted.

The T cell exhaustion phenotype was also identified in other subsets of CD45^−^CXCR5^−^CD8^+^ T cells, such as Treg, Tc1, Tc2, and Tc17 cells, in our study. One observation that deserves attention was the increased expression of IRs in Tc17 cells, followed by that in Tc1 cells. Tc17 cells are characterized by the production of IL17 and the absence of lytic function [10, 11]. Many studies have demonstrated that Tc17 cells play a role in various conditions, such as infection, cancer, and autoimmune inflammation, as documented in both humans and animal models [32–34]. Tc17 promotes terminal exhaustion of CD8^+^ T cells and tumour progression [35]. In addition, Tc17 cells are depleted in HIV infection [36], and dysfunction of activated Tc17 cells is particularly associated with the expression of PD1 [37]. In our study, we found higher expression of IRs, such as PD1 and TIM3, on CD45^−^Tc17 cells in HBV carriers than in healthy volunteers. Another study indicated that Tc17 cells exhibit a memory phenotype (CD28^+^CD45RA^−^) [38] and serve as a self-renewing reservoir of cells that continuously give rise to Tc1-like cells [39]. To identify the function of the two subsets (Tfc and Tc17) with higher expression of IRs, we further investigated the function of T cell subsets after PMA/ionomycin stimulation. The results indicated that the Tfc subset retained high function, while the Tc17 subset exhibited suppression of function. A previous study focused on non-small-cell lung cancer also similarly found that CXCR5^+^ Tfc-like cells rapidly gained a polyfunctional effector phenotype by producing the cytokines TNF-α, IFN-γ, and IL-2 after hours of PMA/ionomycin stimulation [40]. Other researchers have indicated that CXCR5^+^ Tfc cells are armed for effector functions while retaining features of memory cells and express molecules that are candidates for immunotherapeutic intervention, such as PD1 or TIM3 [12]. We speculate that the Tfc subset might be the precursor of exhausted T cells that develop early during infection and are the first to acquire features of exhaustion but sustain and retain some functional properties during chronic infection. A recent study demonstrated that these precursors of exhausted T cells preserve their metabolism to retain long-term functionality, allowing them to sustain T cell responses during chronic infection [41]. Based on the above observations, our findings demonstrate that the CXCR5^+^ Tfc and CXCR5^−^ Tc17 subsets were the main exhausted subsets during chronic HBV infection, while the Tfc subset maintained higher effector functions, and these findings warrant further mechanistic research.

Our study also has some limitations. First, our study was a single-centre investigation in China, and the sample size was relatively small. The findings need to be confirmed in a large multicentre prospective study. Second, the identification of exhausted T cells in HBV carriers was based on the expression of inhibitory receptors and effector cytokines and relied on peripheral T cell profiling due to practical limitations. However, comprehensive analysis of additional features of T cell exhaustion and its function by RNA sequencing combined with profiling of inhibitory receptor expression is still in progress. Third, additional studies performed in mice are warranted in the near future.

## Conclusion

In summary, we herein describe the heterogeneity of the exhausted CD8^+^ T cell subsets in HBV carriers. The CXCR5^+^ Tfc subset was the main exhausted subset but possessed some effector functions during chronic HBV infection. The identification of these unique subsets may contribute to a better understanding of CD8^+^ T cells and provide a potential immunotherapeutic target in chronic HBV infection.

## Supplementary Information

**Additional file 1.** **Table S1.** List of antibodies used in the study. **Table S2.** List of primer sequences used for RT–PCR. **Table S3.** Correlations between the subsets of exhausted Tfc/Tc17 cells and the serum level of HbsAg. **Figure S1.** Gating strategy in CD8^+^ T cell subsets. **A**: gating strategy to detect inhibitory receptors expressed in CD8^+^ T cell subsets; **B**: gating strategy to detect effector cytokines expressed in CD8^+^ T cell subsets. **Figure S2.** Additional data of CD8^+^ T cells in HCs and HBV carriers. **A**: multiple inhibitory receptor expression on CD8^+^ T cells in HCs and HBV carriers; **B**: correlations between the frequencies of PD^+^ or TIM3^+^ Tfc/Tc17 cells and the serum level of TNF-α; **C**: correlations between the frequencies of PD^+^ or TIM3^+^ Tfc/Tc17 cells and the serum level of IFN-γ; **D**-**E**: the production of effector cytokines in Tc/Tfc subsets in HCs (n = 15) and HBV carriers (n = 15); **E**: the production of cytokines in the Tc subset in HBV carriers. HCs: healthy controls, HBV: hepatitis B virus, Tfc: follicular cytotoxic T cells (CXCR5^+^FOXP3^-^), Tc: cytotoxic T cells (CXCR5^-^FOXP3^-^); data are presented as the means ± SEs; *P < 0.01, **P < 0.01.

## Data Availability

All datasets generated for this study are included in the manuscript or the Additional file 1.

## Abbreviations

HBV: Hepatitis B virus
HCs: Healthy controls
PBMCs: Peripheral blood mononuclear cells
Tfc: Follicular cytotoxic T
Treg: Regulatory T
PD1: Programmed cell death protein 1
TIM3: T cell immunoglobulin and mucin domain 3
CTLA4: Cytotoxic T lymphocyte antigen 4
LAG3: Lymphocyte-activation gene 3
IFN-γ: Interferon gamma
TNF-α: Tumour necrosis factor α
CXCR5: C-X-C motif chemokine receptor type 5
