# Supplementary material for: Exhausted phenotype of circulating CD8+ T cell subsets in hepatitis B virus carriers
Source: BMC Immunol. 2022 Apr 20;23:18. doi: 10.1186/s12865-022-00488-2 (PMC9022260; doi:10.1186/s12865-022-00488-2)
Supplement: Supplementary file 1 — Additional file 1. Table S1. List of antibodies used in the study. Table S2. List of primer sequences used for RT–PCR. Table S3. Correlations between the subsets of exhausted Tfc/Tc17 cells and the serum level of HbsAg. Figure S1. Gating strategy in CD8+ T cell subsets. A: gating strategy to detect inhibitory receptors expressed in CD8+ T cell subsets; B: gating strategy to detect effector cytokines expressed in CD8+ T cell subsets. Figure S2. Additional data of CD8+ T cells in HCs and HBV carriers. A: multiple inhibitory receptor expression on CD8+ T cells in HCs and HBV carriers; B: correlations between the frequencies of PD+ or TIM3+ Tfc/Tc17 cells and the serum level of TNF-α; C: correlations between the frequencies of PD+ or TIM3+ Tfc/Tc17 cells and the serum level of IFN-γ; D-E: the production of effector cytokines in Tc/Tfc subsets in HCs (n = 15) and HBV carriers (n = 15); E: the production of cytokines in the Tc subset in HBV carriers. HCs: healthy controls, HBV: hepatitis B virus, Tfc: follicular cytotoxic T cells (CXCR5+FOXP3-), Tc: cytotoxic T cells (CXCR5-FOXP3-); data are presented as the means ± SEs; *P < 0.01, **P < 0.01. [file 12865_2022_488_MOESM1_ESM.docx]

| Specificity | Fluorochrome | Manufacturer | Cat. # |
| --- | --- | --- | --- |
| CD4 | Kromw orange | Beckman | A96417 |
| CD8 | Alexa Fluor 700 | Beckman | B49181 |
| CD45RA | Alexa Fluor 750 | Beckman | B49194 |
| CXCR5 | Pacific blue^TM^ | BioLegend | 356918 |
| CD25 | ECD | Beckman | 6607112 |
| PD1 | PerCP/Cy5-5 | BioLegend | 329914 |
| TIM3 | APC | BioLegend | 345012 |
| LAG3 | Brillian violet 650^TM^ | BioLegend | 369316 |
| CTLA4 | PE/Cy7 | BioLegend | 369614 |
| CXCR3 | FITC | BioLegend | 353704 |
| CCR6 | PE | BioLegend | 353410 |
| CD4 | Alexa Fluor® 700 | BioLegend | 300526 |
| CD8 | APC/Cyanine7 | BioLegend | 344714 |
| CD45RA | Brilliant Violet 510™ | BioLegend | 304141 |
| CXCR5 | Brilliant Violet 605™ | BioLegend | 356930 |
| FOXP3 | Brilliant Violet 421™ | BioLegend | 320124 |
| CD107a | Brilliant Violet 650™ | BioLegend | 328638 |
| IFN-γ | PE/Cyanine7 | BioLegend | 502528 |
| TNF-α | Brilliant Violet 785™ | BioLegend | 502948 |
| Granzyme B | APC | BioLegend | 372204 |

Table S1. List of antibodies used in the study

Table S2. List of primer sequences used for RT–PCR

| Gene | Forward primers | Reverse primer |
| --- | --- | --- |
| TNFα | 5’-CTCTTCTGCCTGCTGCACTTTG-3’ | 5’-ATGGGCTACAGGCTTGTCACTC-3’ |
| IFNγ | 5’-GAGTGTGGAGACCATCAAGGAAG-3’ | 5’-TGCTTTGCGTTGGACATTCAAGTC-3’ |
| TOX | 5’-CGCTACCTTTGGCGAAGTCTCT-3’ | 5’-CTGGCTCTGTATGCTGCGAGTT-3’ |
| PD1 | 5’-AAGGCGCAGATCAAAGAGAGCC-3’ | 5’-CAACCACCAGGGTTTGGAACTG-3’ |
| TIM3 | 5’-GACTCTAGCAGACAGTGGGATC-3’ | 5’-GGTGGTAAGCATCCTTGGAAAGG-3’ |
| CTLA4 | 5’-ACGGGACTCTACATCTGCAAGG-3’ | 5’-GGAGGAAGTCAGAATCTGGGCA-3’ |

*The primer sequences used for RT-PCR was provided by the website of https://www.origene.com/

Table S3. Correlations between the subsets of exhausted Tfc/Tc17 cells and the serum level of HbsAg

| Correlation (n = 31) | Spearman r | P (two-tailed) |
| --- | --- | --- |
| HBsAg vs. TIM3+Tfc | -0.1657 | 0.3729 |
| HBsAg vs. PD1+Tfc | -0.09516 | 0.6106 |
| HBsAg vs. CTLA4+Tfc | -0.06333 | 0.7350 |
| HBsAg vs. LAG3+Tfc | 0.2307 | 0.2118 |
| HBsAg vs. TIM3+Tc17 | -0.3036 | 0.0968 |
| HBsAg vs. PD1+Tc17 | -0.2802 | 0.1268 |
| HBsAg vs. CTLA4+Tc17 | -0.07258 | 0.6980 |
| HBsAg vs. LAG3+Tc17 | -0.09678 | 0.6045 |


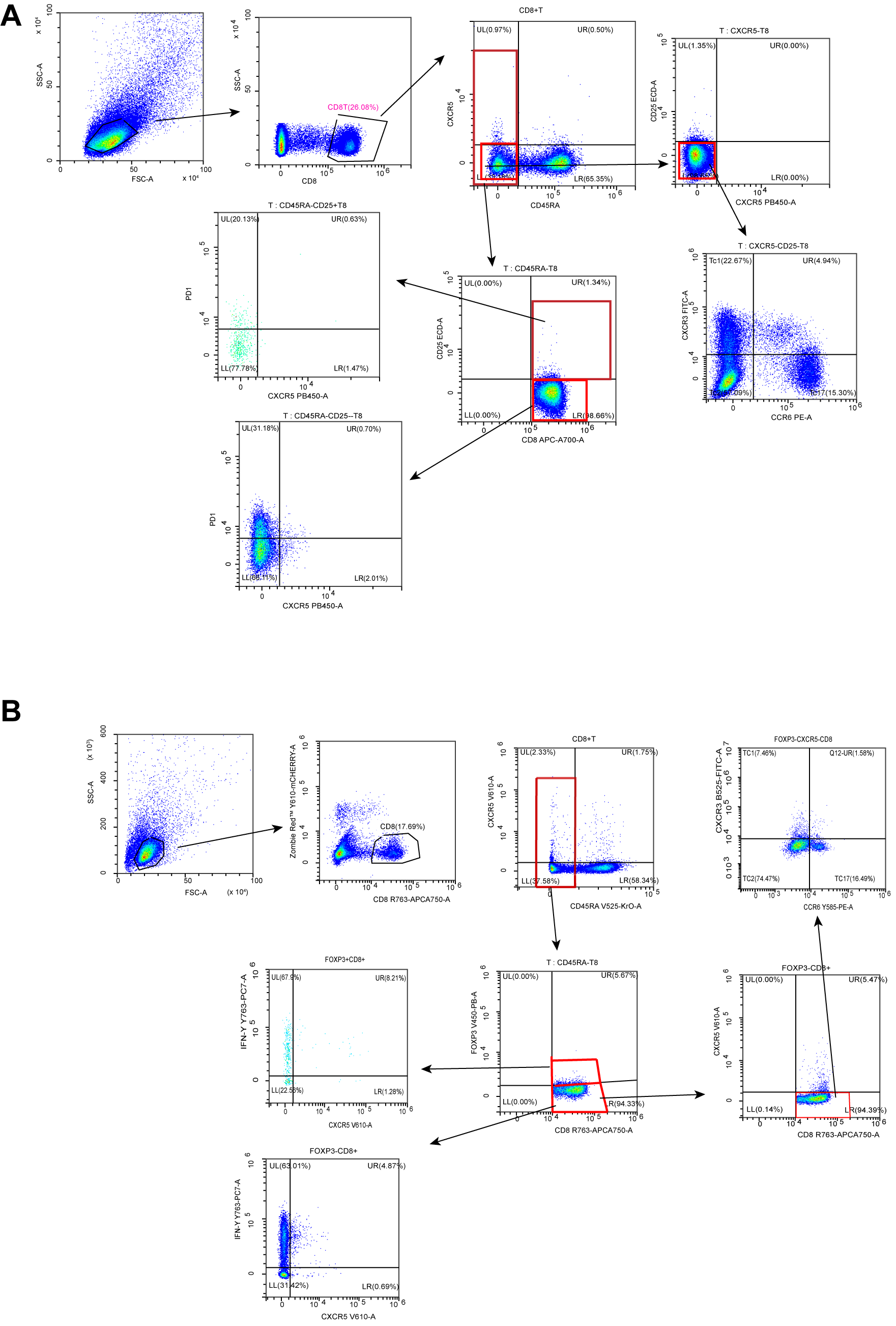


Fig S1. Gating strategy in CD8^+^ T cell subsets. A: gating strategy to detect inhibitory receptors expressed in CD8^+^ T cell subsets; B: gating strategy to detect effector cytokines expressed in CD8^+^ T cell subsets.


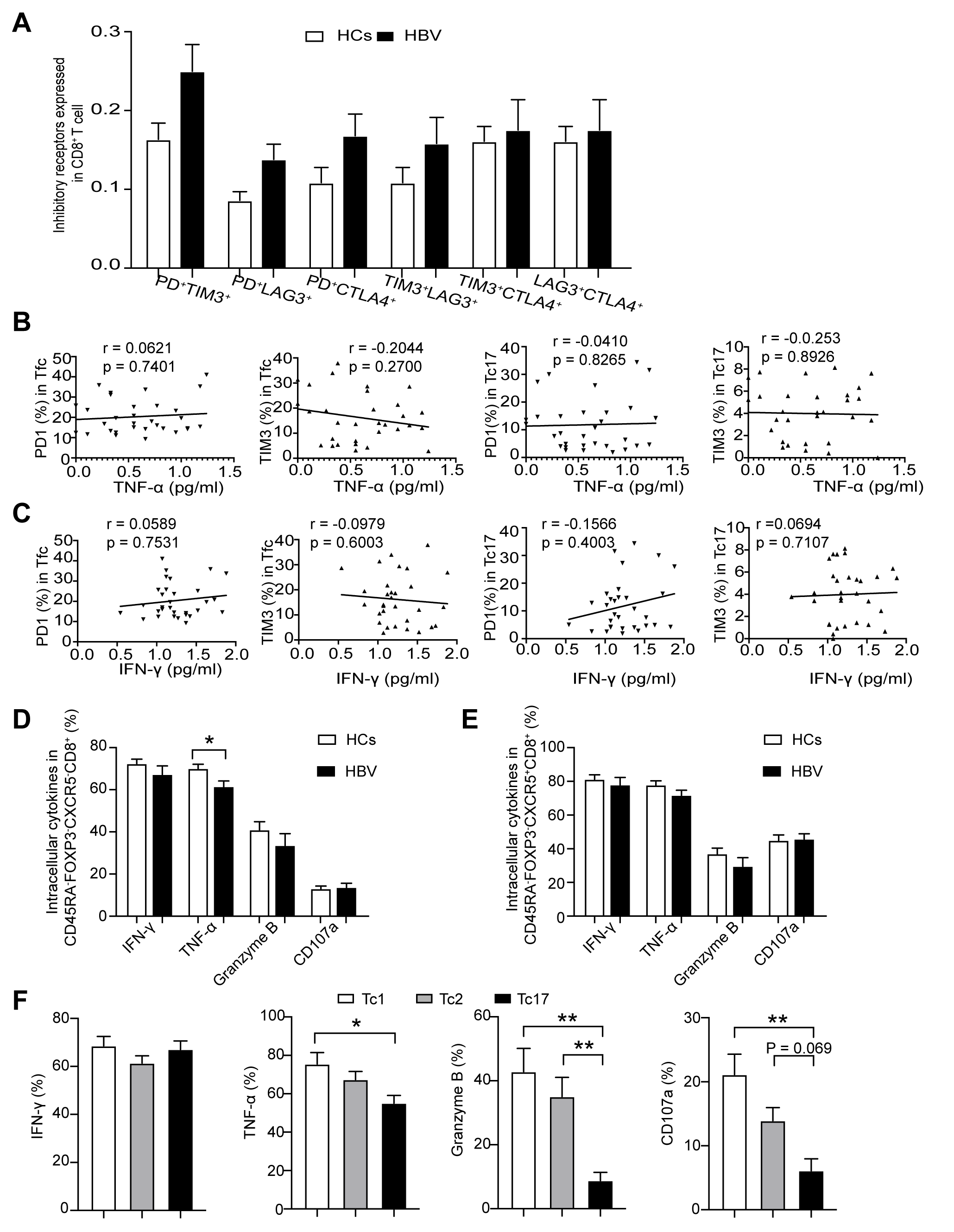
Fig S2. Additional data of CD8^+^ T cells in HCs and HBV carriers. A: multiple inhibitory receptor expression on CD8^+^ T cells in HCs and HBV carriers; B: correlations between the frequencies of PD^+^ or TIM3^+^ Tfc/Tc17 cells and the serum level of TNF-α; C: correlations between the frequencies of PD^+^ or TIM3^+^ Tfc/Tc17 cells and the serum level of IFN-γ; D-E: the production of effector cytokines in Tc/Tfc subsets in HCs (n = 15) and HBV carriers (n = 15); F: the production of cytokines in the Tc subsets in HBV carriers. HCs: healthy controls, HBV: hepatitis B virus, Tfc: follicular cytotoxic T cells (CXCR5^+^FOXP3^-^), Tc: cytotoxic T cells (CXCR5^-^FOXP3^-^); data are presented as the means ± SEs; **P* < 0.01, ***P* < 0.01.
